# Biomolecular condensate assembly of nArgBP2 tunes its functionality to manifest the structural plasticity of dendritic spines

**DOI:** 10.1038/s12276-022-00918-6

**Published:** 2023-01-04

**Authors:** Eunji Cho, Sang-Eun Lee, Unghwi Lee, Yuna Goh, Seonyoung Jeong, Junyoung Choi, Won-Ki Jeong, Sunghoe Chang

**Affiliations:** 1 Department of Physiology and Biomedical Sciences, Seoul National University College of Medicine, Seoul, 03080 South Korea; 2 UK Dementia Research Institute, University College London, Gower St, London, WC1E 6BT UK; 3 School of Electrical and Computer Engineering, Ulsan National Institute of Science and Technology, Ulsan, 44919 South Korea; 4 Department of Computer Science and Engineering, Korea University, Seoul, 02841 South Korea

**Keywords:** Cellular neuroscience, Spine regulation and structure

## Abstract

nArgBP2, a candidate gene for intellectual disability, is a postsynaptic protein critical for dendritic spine development and morphogenesis, and its knockdown (KD) in developing neurons severely impairs spine-bearing excitatory synapse formation. Surprisingly, nArgBP2 KD in mature neurons did not cause morphological defects in the existing spines at rest, raising questions of how it functions in mature neurons. We found that unlike its inaction at rest, nArgBP2 KD completely inhibited the enlargement of dendritic spines during chemically induced long-term potentiation (cLTP) in mature neurons. We further found that nArgBP2 forms condensates in dendritic spines and that these condensates are dispersed by cLTP, which spatiotemporally coincides with spine head enlargement. Condensates with CaMKII phosphorylation-deficient mutant or CaMKII inhibition are neither dispersed nor accompanied by spine enlargement during cLTP. We found that nArgBP2 condensates in spines exhibited liquid-like properties, and in heterologous and in vitro expression systems, nArgBP2 undergoes liquid-liquid phase separation via multivalent intermolecular interactions between SH3 domains and proline-rich domains. It also forms coacervates with CaMKIIα, which is rapidly dissembled by calcium/CaMKIIα-dependent phosphorylation. We further showed that the interaction between nArgBP2 and WAVE1 competes with nArgBP2 phase separation and that blocking the nArgBP2-WAVE1 interaction prevents spine enlargement during cLTP. Together, our results suggest that nArgBP2 at rest is confined to the condensates but is released by CaMKIIα-mediated phosphorylation during synaptic plasticity, which regulates its timely interaction with WAVE1 to induce spine head enlargement in mature neurons.

## Neuroscience: Synapse protein dynamics linked to brain connectivity

A critical protein forms transient assembles inside nerve cells to control adaptive brain responses, a process that, when dysfunctional, may contribute to intellectual disability. Sunghoe Chang of Seoul National University College of Medicine, South Korea, and colleagues showed that when neurons are at rest, a synaptic scaffolding protein called nArgBP2, a key regulator of excitatory synapse formation, accumulates inside liquid-like droplets known as biomolecular condensates and remains dormant. In response to neuronal activation, however, these condensates break up, freeing nArgBP2 to interact with other proteins that promote the enlargement of dendritic spines, small protrusions that receive most of the excitatory synaptic inputs, a critical step in learning and memory. Aberrant regulation of this process impairs the brain’s ability to respond to electrochemical cues, which could help explain why a deficiency in nArgBP2 is associated with mood disorders and intellectual impairment.

## Introduction

Dendritic spines are tiny protrusions arising from dendrites, in which most excitatory synapses are located^1^. Activity-dependent maintenance or elimination of dendritic spines is critical for remodeling neural circuits during development and synaptic plasticity^2^. Dendritic spines are highly enriched in F-actin; thus, the activity of actin-regulating proteins has a major influence on spine morphology and is closely related to various psychiatric disorders.

(Neural) Arg/c-Abl kinase binding protein 2 (ArgBP2/nArgBP2), also known as sorbin and SH3 domain-containing 2 (SORBS2), is located on chromosome 4q35.1 in humans and has been linked to intellectual disability (ID)^3^. ArgBP2/nArgBP2 belongs to an adaptor protein family that includes vinexin and CAP/ponsin, which are thought to participate in the regulation of cell adhesion, actin cytoskeleton organization, and growth factor receptor signaling^4^. This family is characterized by a sorbin homology (SoHo) domain in the NH_2_-terminal region and three SH3 domains in the COOH-terminal region^4–6^. Neuron-specific nArgBP2 was found to localize to synapses interacting with synapse-associated protein 90/postsynaptic density (PSD)-95-associated protein (SAPAP)^4,7^. The SH3 domains of nArgBP2 bind to synaptojanin 1/2, dynamin 1/2, and Wiskott–Aldrich syndrome protein-family verprolin homologous protein (WAVE) 1/2, and knocking down ArgBP2/nArgBP2 expression in astrocytes induces the redistribution of focal adhesion proteins and an increase of peripheral actin ruffles^7,8^.

*nArgBP2* knock-out (KO) mice have been reported to display manic/bipolar-like behavior resembling many aspects of the symptoms of patients with bipolar disorder (BD)^9^. In addition, copy number variation of nArgBP2 has been linked to ID^10^, and deletion of *nArgBP2* led to impaired dendritic complexity accompanied by ID-like behavioral deficits^11^. We found that nArgBP2 KD in developing neurons causes a dramatic change in spine morphology and a selective inhibition of excitatory spine-synapse formation, causing an excitatory-inhibitory synaptic imbalance (E/I imbalance)^12^.

nArgBP2 expression remains at high levels until mature stages, implying its role in mature neurons, but despite having such profound effects on developing neurons, we found that nArgBP2 KD in mature neurons did not result in any morphological defects in existing dendritic spines at rest. Through a series of experiments using living neurons and heterologous and in vitro expression systems, we demonstrated that nArgBP2 at rest is sequestered in liquid condensates and is released upon CaMKIIα-mediated phosphorylation during LTP, which regulates its timely interaction with WAVE1 to induce the enlargement of dendritic spines. Together, our results can provide important mechanistic insight into nArgBP2 function in the spatiotemporal regulation of the structural plasticity of dendritic spines and suggest the possibility that defects in the phase-separating behavior of nArgBP2 may be related to the synaptic dysfunction observed in ID.

## Materials and methods

Animal experiments were approved by the Institute of Animal Care and Use Committee (IACUC, Approval ID number: SNU-100930-5) of Seoul National University, Korea. All experiments were carried out under approved guidelines and regulations.

### DNA constructs and antibodies

RNA interference-mediated nArgBP2 knockdown was carried out by expressing small hairpin RNA (shRNA) duplexes in the pSiren-U6-mRFP vector (Clontech, Palo Alto, CA) as previously described^12^. Silent mutations within the shRNA targeting sequence (C593T, G596A, and A599G) in EGFP-nArgBP2 (EGFP-nArgBP2-*res*) were generated as previously described^12^. EGFP-nArgBP2_959-1196_-3S3A and 3S3D were constructed using an EZchange™ Multi Site-directed Mutagenesis kit (Enzynomics, Daejeon, South Korea). CaMKIIα-SBFP2 was constructed by subcloning CaMKIIα from CaMKIIα-Venus (Addgene) by PCR in the SBFP2-N1 vector. EGFP-mito-nArgBP2_959-1110_-2P2A was derived from EGFP-mito-nArgBP2_959-1110_ by introducing P1027A and P1030A mutations, which have the first and second SH3 domains of nArgBP2 with a mitochondrial localization signal. All constructs were verified by DNA sequencing. The antibodies used in this study, including working dilutions, are listed in Supplementary Table 1.

### Primary neuron culture and transfection

Primary rat hippocampal neurons derived from embryonic Day 18 Sprague Dawley fetal rats of either sex were prepared as described previously^13^. Briefly, hippocampi were dissected, dissociated with papain (Worthington Biochemical Corporation, Lakewood, NJ), resuspended in minimal Eagle’s medium (MEM, Invitrogen) supplemented with 0.6% glucose, 1 mM pyruvate, 2 mM L-glutamine, and 10% fetal bovine serum (HyClone, South Logan, UT), and plated on poly-D-lysine-coated glass coverslips in 60 mm Petri dishes. 4 h after plating, the medium was replaced with a neurobasal medium (Invitrogen) supplemented with 2% NS21 and 0.5 mM L-glutamine. Neurons were transfected by a modified calcium-phosphate method as previously described^13^.

### Chemically induced long-term potentiation of hippocampal neurons

Transfected mature neurons (DIV 21) were preincubated with 50 µM AP5 for 48 h in neurobasal media and transferred to a magnesium-free Tyrode’s solution (119 mM NaCl, 2.5 mM KCl, 2 mM CaCl_2_, 2 mM MgCl_2_, 25 mM HEPES, pH 7.4 and 30 mM glucose). Images were acquired at 10 s intervals, and after the second acquisition, cLTP-inducing solution (200 µM glycine, 20 µM bicuculline, 1 µM strychnine, 0.5 µM TTX in Mg^2+^-free Tyrode) was added for 3 min. The solution was changed to magnesium-free Tyrode’s solution, and the cells were imaged for an additional 4 min at 10 s intervals.

### Classification of dendritic spines

Transfected neurons were rinsed with Tyrode’s solution and fixed with 4% paraformaldehyde (PFA) in 4% sucrose-containing 0.1 M phosphate-buffered saline (PBS) (pH 7.3) for 10 min at RT and then washed with PBS. Images were acquired with an inverted microscope (IX71, Olympus, Tokyo, Japan) equipped with an sCMOS camera (Zyla-5.5-CL3, Andor Technology, Belfast, Ireland). Well-branched pyramidal neurons were randomly selected, and the analysis was performed in a single-blinded manner. To categorize the spines, fluorescent images were imported into NeuronStudio^14^ for automated detection of dendrites and spines.

### 3D-structured illumination microscopy (SIM) imaging and data processing

After cLTP induction, neurons mounted in a chamber were imaged using an N-SIM microscope (ECLIPSE Ti-E, Nikon, Tokyo, Japan) equipped with an oil immersion TIRF objective lens (Apo TIRF 100× N.A. 1.49), and an EMCCD camera (iXon DU-897, Andor Technology). The lateral and axial resolutions measured using 100 nm diameter beads are 115 and 269 nm, respectively, in 3D-SIM mode. The acquired datasets, comprising 48 axial sections of 512 × 512 pixels, were computationally reconstructed using the reconstruction stack algorithm of NIS-Elements AR software (Nikon). The voxel size of the reconstructed images was 32 nm in the *x*- and *y*-dimensions and 120 nm in the *z*-dimension, with 16-bit depth. The reconstructed SIM image stacks were processed with DXplorer^15^.

### Shape factor analysis

Changes in spine morphology were assessed from time-lapse images of shRNA-nArgBP2 with or without nArgBP2-*res* using the “Shape Descriptors” plug-in in Fiji. The form factor (*f* = 4π*A*/*p*^2^) was calculated from the perimeter *p* and the area *A* of the object. The value approaches 1 as the spine head is rounder and 0 as the spine head is more irregular or elongated.

### Western blotting and immunoprecipitation

Samples were lysed in 1% Triton-X 100 buffer (20 mM Tris pH 7.5, 137 mM NaCl, 10% glycerol, 1% Triton-X 100, 2 mM EDTA) in the presence of a protease inhibitor mixture (Roche), clarified by centrifugation at 13,200 × *g* for 20 min, and concentrations were measured with a Bicinchoninic acid (BCA) Protein Assay Reagent Kit (ThermoFisher, Waltham, MA). For immunoprecipitation experiments, ionomycin was added before lysis, and lysates were incubated with primary antibody at 4 °C for 2 h and then 2 h after adding Protein A-Sepharose beads (GE Healthcare, Chicago, IL). Then, the samples were separated by SDS‒PAGE and transferred to PVDF membranes (Merck, Burlington, MA). The membranes were blocked for 30 min with 5% (wt/vol) nonfat dry milk in TBST (10 mM Tris⋅HCl pH 7.6, 100 mM NaCl, 0.1% Tween 20) incubated with the primary antibodies overnight at 4 °C and incubated with the corresponding HRP-conjugated secondary antibody for 1 h at room temperature. Chemiluminescence reactions were performed with an AbSignal Western detection kit system (AbClon, Seoul, South Korea) and acquired using an ImageQuant LAS 4000 (GE Healthcare).

### Cell culture and transfection

COS7 cells were cultured at 37 °C in 5% CO_2_ in DMEM (Invitrogen, Carlsbad, CA) supplemented with 10% fetal bovine serum and transfected with constructs using PEI (MW 40000) (Polysciences, Warrington, PA) at a ratio of 1:4 (total DNA (μg) to PEI (μL)).

### Protein purification

All proteins were expressed in *Escherichia coli* BL21 (DE3). Cells were grown at 37 °C in 2xYT medium with ampicillin (50 μg/ml) to an A_600_ of 0.6–0.8, followed by induction with 0.5 mM isopropyl-β-D-thiogalactopyranoside (IPTG) at 37 °C for 4 h or at 16 °C overnight. Cells resuspended in lysis buffer (25 mM HEPES [pH 7.4], 400 mM KCl, 20 mM imidazole, 10% glycerol, 0.5% Triton X-100, 1 mg/ml lysozyme, 0.1 mg/ml DNase I, 1 mM PMSF, protease inhibitor cocktail) were sonicated and rocked at 4 °C for 1 h with 0.5% n-lauroylsarcosine sodium salt. After centrifugation, the supernatant was incubated with Ni-NTA chelating agarose beads (Incospharm, Daejeon, South Korea) at 4 °C. Proteins were eluted with a buffer containing 25 mM HEPES [pH 7.4], 300 mM imidazole, 1 mM DTT, and various concentrations of KCl. All proteins were quantified by SDS‒PAGE.

### In vitro droplet imaging

Protein solutions were injected into a custom-made chamber assembled by attaching a cleaned 18 mm coverslip onto a glass slide. For CaMKIIα in vitro droplet imaging, 1 μM CaMKIIα (Thermo) was incubated in CaMKIIα activation buffer (50 mM Tris, pH 7.5, 10 mM MgCl_2_, 2 mM DTT, 0.1 mM Na_2_EDTA, 2 mM CaCl_2_, 100 μM ATP, 1.2 μM calmodulin) for 10 min and then added to proteins. Images were acquired with a 488 or 561 nm laser using a spinning disk confocal microscope (ECLIPSE Ti-E, Nikon) with an oil immersion objective lens (Plan Apo 60× N.A. 1.40), and a Neo sCMOS camera (Andor Technology) at room temperature. Phase separation was noted by visual inspection and analysis using ImageJ software (NIH).

### Quantification of droplet formation in COS7 cells

Average fluorescence intensity (intensity/cell area) values reflecting the expression level of the fluorescent protein in each cell were measured using ImageJ. The presence of droplets was assessed as follows: we first subtracted the background from each image and applied the threshold function to acquire a binarized image; then, the number of droplets was quantified using the ‘Analyze Particles’ command. Droplets with a size < 0.4 μm^2^ and with circularity <0.8 were excluded [circularity = 4 pi (area/perimeter^2^); 1.0 = a perfect circle]. If the droplet count was < 5, we visually examined the original image to ensure the presence of droplets. The analysis was performed at least three times on a single-blind basis to ensure reproducibility.

### FRAP assays

Experiments were performed using the stimulus-setting menu in the Nikon A1 to control sequential image acquisition using a 60× oil-immersion lens (1.40 N.A.) equipped with a Nikon A1 confocal microscope (Nikon) to accomplish photobleaching of a circular or cylindrical ROI by laser pulse emission. ROIs containing single droplets of COS7 cells were imaged every 5 s. After 5 images had been acquired, the droplet was photobleached for 2.5 s with a 488 nm laser (100%), and fluorescence recovery was imaged at 5 s intervals at 37 °C. Average intensity values of ROI and total image fluorescence were obtained from each FRAP image using Nikon imaging software (NIS-elements). ROI values over time were plotted. Fluorescence intensities in the bleached ROIs were normalized to initial values.

### Fluorescence live-cell imaging

Live cell images were acquired using a spinning disk confocal microscope (Nikon).

#### 1,6-hexanediol treatment

COS7 cells and neurons in Tyrode’s solution were imaged at 5 s intervals and exposed to 3% 1,6-hexanediol (Sigma) in Tyrode’s solution.

#### Ionomycin

Time-lapse images of transfected COS7 cells were acquired for 1 min at 5 s intervals. After the third acquisition, ionomycin (Sigma) was added to a working concentration of 10 µM.

### Statistics

The normality of the data was examined with the Kolmogorov–Smirnov normality test. Student’s two-sample *t-test* was used to compare pairs of independent groups, and one-way ANOVA followed by Tukey’s honest significant difference (HSD) post hoc test was used for multiple conditions. When the normality of data could not be assumed, the Kruskal–Wallis test was used for the nonparametric comparison of multiple groups. Prism 8 (GraphPad Software, San Diego, CA) was used for statistical analysis. The relevant p values are presented in the figure legends, and unless otherwise indicated, data are presented as the means ± s.e.m. (Standard Error of the Mean) or s.d. (Standard deviation), with *n* indicating the number of independent experiments.

## Results

### nArgBP2 KD does not cause any morphological defects in the existing dendritic spines of mature neurons but completely blocks the enlargement of spines during cLTP

**Fig. 1:**
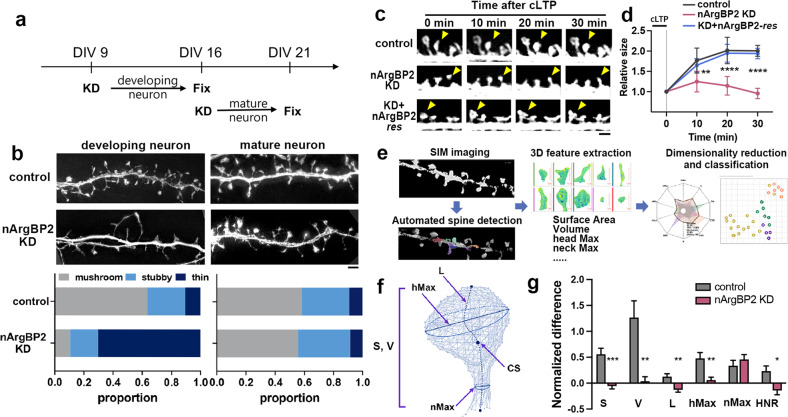
nArgBP2 KD in mature neurons does not result in morphological defects in existing dendritic spines but completely inhibits spine head enlargement during cLTP. **a** Schematic overview of nArgBP2 KD in developing and mature neurons. **b** Representative images of spine morphologies and the proportions of mushroom-shaped, stubby, and thin spines in neurons expressing control or shRNA-nArgBP2 in developing neurons and mature neurons. Developing neurons were transfected at DIV 9 and fixed at DIV 16, whereas mature neurons were transfected at DIV 16 and fixed at DIV 21. Scale bar: 2 μm. The proportion of each is shown above the bar. **c** Representative time-lapse images of spines in control and nArgBP2 KD neurons during cLTP induction. Neurons at DIV 16 were transfected with shRNA-nArgBP2 alone or combined with EGFP-nArgBP2-*res* and were imaged at DIV 21. Note the gradual enlargement of the spine heads in control and KD + EGFP-nArgBP2-*res* neurons but not in KD neurons. The yellow arrowheads indicate the spine heads. Scale bars: 2 μm. **d** The relative size of spine heads in control and nArgBP2 KD neurons after cLTP, normalized to the initial value. *n* = 9 for control, 11 for KD, and 7 for KD + EGFP-nArgBP2-*res* (one-way ANOVA followed by Tukey’s HSD test). *p < 0.05; **p < 0.01. Error bars indicate the means ± s.e.m.s. **e** Schematic diagram of the process of 3D geometry analysis of dendritic spines using DXplorer. The left panel shows the acquisition of 3D-SIM images and automatic detection of dendritic spines. 3D geometrical features were extracted and calculated. The datasets were further analyzed and classified by dimensionality reduction and machine-learning-based automatic classification. **f** Representative morphological features extracted from the 3D mesh of dendritic spines. S, Surface; V, Volume; L, length of spine; hMax, the maximum diameter of the spine head; nMax, the maximum diameter of the spine neck; CS, the centroid of the spine. **g** Normalized differences in morphological features of spines between pre-and post cLTP in control and nArgBP2 KD neurons. HNR, the ratio of head to the neck (HNR = hMax/nMax); *n* = 39 for control, 34 for KD. *p < 0.05; **p < 0.01; ***p < 0.001. Student’s *t*-test; error bars indicate the mean ± s.d.

Knockdown (KD) of nArgBP2 in developing neurons caused drastic inhibition of the formation of mushroom-shaped dendritic spines and selective inhibition of excitatory spine-synapse formation (Fig. 1a, b)^12^. Despite having such profound effects on developing neurons, however, nArgBP2 KD in mature hippocampal neurons (KD at DIV 16 and observed at DIV 21; Supplementary Fig. 1) did not result in any morphological defects in the existing dendritic spines (Fig. 1b).

We hypothesized that nArgBP2 might function when neurons undergo dynamic structural remodeling, such as during development or synaptic plasticity^2^. To test this possibility, we induced glycine-induced chemical long-term potentiation (cLTP) in cultured mature neurons, which is known to activate synaptic NMDA receptors and induce the enlargement of dendritic spines^16,17^. We found that cLTP induced a significant increase in the size of the spine heads in control neurons, while it failed to do so in nArgBP2 KD neurons (Fig. 1c, d). In control neurons, the relative size of dendritic spines gradually increased for 30 min after induction of cLTP, whereas in KD neurons, it instead decreased (2.00 ± 0.13 for control, 0.96 ± 0.13 for KD). The expression levels of the NMDA receptors GluN1 and GluN2 were not altered with nArgBP2 KD (Supplementary Fig. 2). The defect in spine enlargement was fully rescued by the expression of shRNA-resistant nArgBP2 (EGFP-nArgBP2-*res*), ruling out the off-target effects of shRNA expression (Fig. 1c, d).

To more accurately analyze the alteration in spine morphology in 3 dimensions (3D), we used DXplorer, a machine-learning-based 3D spine morphology analysis program that we recently developed^15^. We first acquired 3D spine images before and after cLTP using a SIM and extracted a set of morphological high-dimensional features from the 3D meshes of dendritic spines (Fig. 1e). We then measured the ratio of these values before and after cLTP (Post/Pre, Fig. 1f, g). The relative surface area after cLTP increased significantly in the control spines but decreased in the KD spines (S; 1.47 ± 0.09 for control, 0.93 ± 0.06 for shRNA, Fig. 1g). The volume of control spines doubled after cLTP but remained the same in KD spines (2.04 ± 0.24 for control, 1.01 ± 0.09 for shRNA, Fig. 1g). While the maximum neck diameters in control and KD neurons were similar, the ratio of maximum head diameter to neck diameter increased significantly in controls, indicating that nArgBP2 functions as a spine-head expander (Fig. 1g). Thus, unlike its inaction at rest, nArgBP2 KD exerts its inhibitory effect when challenged with cLTP, in which circumstance it completely suppresses the enlargement of the spine heads in mature neurons.

### nArgBP2 forms condensates in dendritic spines and these condensates are dispersed by CaMKIIα-mediated phosphorylation during chemical-LTP, which spatiotemporally coincides with spine head enlargement

We next sought to determine the underlying mechanism of nArgBP2-mediated spine enlargement during cLTP. To monitor nArgBP2 behaviors in living neurons, we first knocked down endogenous nArgBP2 and expressed the shRNA-resistant form of nArgBP2-*res* in a KD background. Thus, we expect to replace endogenous nArgBP2 with EGFP-nArgBP2 and circumvent overexpression artifacts. We confirmed that the expression of EGFP-nArgBP2-*res* in a KD background resulted in a comparable expression level to the endogenous nArgBP2 level (Supplementary Fig. 3), and subsequent experiments in living neurons were performed using expression of shRNA-resistant forms in the background of KD (hereinafter only the construct name is referred to).

**Fig. 2:**
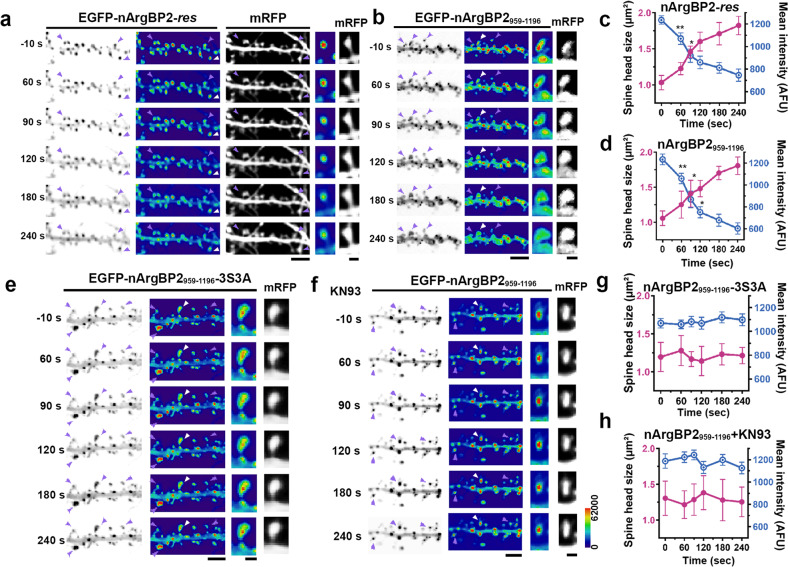
nArgBP2 forms condensates in dendritic spines, and these condensates are dispersed by CaMKIIα-mediated phosphorylation during chemical-LTP, which spatiotemporally coincides with spine head enlargement. Hippocampal neurons were transfected with EGFP-nArgBP2-*res*_,_ EGFP-nArgBP2_959-1196,_ or EGFP-nArgBP2_959-1196_-3S3A and mRFP-tagged shRNA-nArgBP2 to exclude the effect of endogenous nArgBP2. Representative time-lapse inverted images and pseudocolored images of hippocampal neurons transfected with EGFP-nArgBP2-*res* (**a**), EGFP-nArgBP2_959-1196_ (**b**), EGFP-nArgBP2_959-1196_-3S3A (**e**), or EGFP-nArgBP2_959-1196_ with KN93 treatment (**f**) upon cLTP stimulation. The third columns show magnified pseudocolored views of the spines indicated by white arrowheads. The last columns show the mRFP signal in the spine cytosol used as a volume marker. Scale bars: 10 and 1 μm, respectively. Plots of time-dependent changes in single spine head size and mean intensity of EGFP-nArgBP2-*res* (**c**), EGFP-nArgBP2_959-1196_ (**d**), EGFP-nArgBP2_959-1196_-3S3A (**g**), and EGFP-nArgBP2_959-1196_ with KN93 treatment (**h**) spines after cLTP. Both EGFP-nArgBP2-*res* and EGP-nArgBP2_959-1196_ but not EGFP-nArgBP2_959-1196_-3S3A and EGFP-nArgBP2_959-1196_ with KN93 treatment droplets in the dendritic spine dispersed rapidly during cLTP, leading to spine enlargement. See Methods for more details. *n* = 10; *p < 0.05; **p < 0.01; the spines and droplets in the third columns of **a**, **b**, **e**, and **f** were used for the analysis.

We found that EGFP-full-length nArgBP2 formed condensates that are located mostly in dendritic spines and partially in the shafts, consistent with previous studies on nArgBP2 localization in dendritic spines^11,12^. Then, we induced glycine-induced cLTP in hippocampal neurons at DIV 21 (Fig. 2). We found that the EGFP-nArgBP2 condensates rapidly dispersed into the cytosol of the dendritic spines within a few minutes of cLTP induction, while those in the dendritic shafts remained unaffected (Fig. 2a). More importantly, we found that condensate dispersion was accompanied by the expansion of the dendritic spine heads, and there was a spatiotemporal correlation between them (Fig. 2a, c).

We further found that the above phenomena with full-length nArgBP2 were fully recapitulated by its SH3 domain mutant, nArgBP2_959-1196_. As observed with full-length EGFP-nArgBP2_959-1196,_ condensates rapidly dispersed into the cytosol of the dendritic spines within a few minutes of cLTP induction (Fig. 2b). Furthermore, we found that EGFP-nArgBP2_959-1196_ condensate dispersion accompanies spine enlargement (Fig. 2b, d). These results are consistent with our previous results that SH3 domains are the self-functional modules that mediate the structural remodeling of dendritic spines^12^. The overexpression of full-length nArgBP2 often caused large actin aggregates, which is consistent with previous reports that nArgBP2 overexpression induced the coalescence of F-actin into large aggregates due to the N-terminal SoHo domain^7,18^. To avoid this complication, we decided to use nArgBP2_959-1196_ in further experiments. We believe this alternative is legitimate given that the SH3 domain is the functional domain that drives structural remodeling in spines and behaves similarly to the full-length protein (Fig. 2a–d).

### Dispersion of nArgBP2 condensates by CaMKIIα-mediated phosphorylation

Calcium/calmodulin-dependent protein kinase IIα (CaMKIIα) is a key effector controlling spine enlargement and synaptic strength during LTP in vitro and in vivo^17,19,20^. nArgBP2 contains several putative CaMKIIα-dependent phosphorylation sites (see below), and thus, CaMKIIα could be an effector to dissolve nArgBP2 condensates upon cLTP induction.

nArgBP2_959-1196_ is predicted to contain a consensus sequence for phosphorylation by CaMKII (RXXS/T) at S1119 and two nonconsensus sequences at S975 and S1179^21^. Since all three residues are conserved among rat, human, and mouse SORBS2 (Supplementary Fig. 4a), we decided to construct a phospho-deficient mutant in which S975, S1119, and S1179 were modified to alanine (3S3A) (Supplementary Fig. 4b). We found that phospho-deficient EGFP-nArgBP2_959-1196_-3S3A also formed condensates in dendritic spines but remained assembled during cLTP, and no spine expansion occurred (Fig. 2e, g). Accordingly, the CaMKII inhibitor KN93 completely abolished EGFP-nArgBP2_959-1196_ dispersion and spine expansion (Fig. 2f, h). These results indicate that CaMKIIα-mediated phosphorylation is required for the dispersion of nArgBP2 condensates and spine enlargement during cLTP.

### Phase separation of nArgBP2 in heterologous and in vitro expression systems

The rapid CaMKIIα-dependent dispersion of ArgBP2 condensates during cLTP suggests that these condensates are not solid-like aggregates but rather resemble liquid-like condensates caused by liquid–liquid phase separation (LLPS)^22,23^. Indeed, nArgBP2 condensates in spines spontaneously fused with each other (Supplementary Fig. 5a, b) and were dispersed by 3% 1,6-hexanediol (1,6-HD), an alcohol known to disperse a variety of biomolecular condensates formed by LLPS via a mechanism that involves its hydrophobicity^24,25^ (Supplementary Fig. 5c–g). We further found that endogenous nArgBP2 also formed condensates in spines that were dispersed by 1,6-HD (Supplementary Fig. 6).

**Fig. 3:**
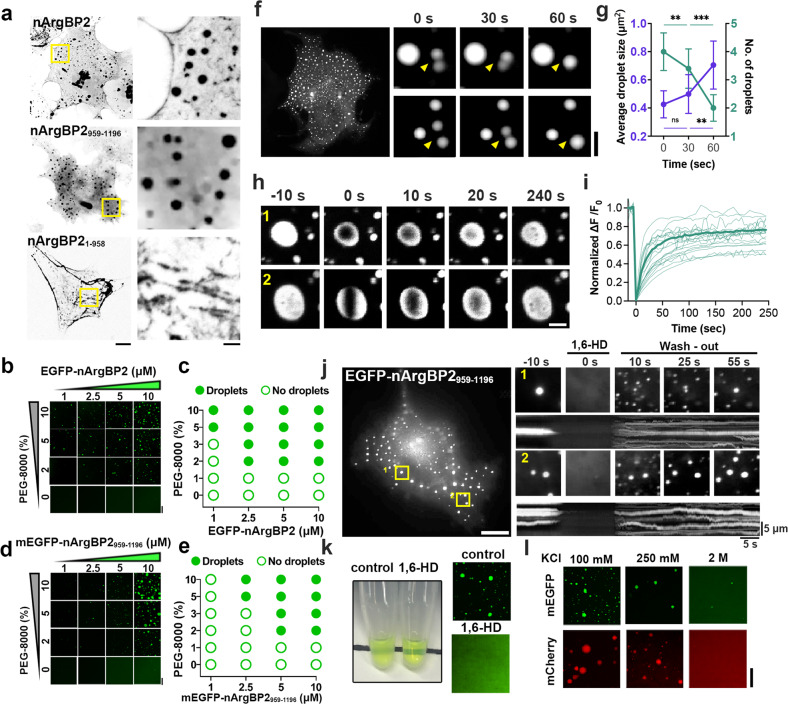
Biomolecular condensate assembly of nArgBP2 in heterologous and in vitro expression systems. **a** Representative images of EGFP-tagged full-length nArgBP2, nArgBP2_959-1196_, and SH3 deletion mutant expressed in COS7 cells imaged 48 h after transfection. Scale bar: 10 µm. **b**–**e** Fluorescence images of purified EGFP-nArgBP2 or mEGFP-nArgBP2_959-1196_ over a range of protein concentrations with or without the crowding agent PEG-8000. Note that the formation of droplets is facilitated, and the size of droplets increases as the concentration of protein or PEG-8000 increases. Scale bar: 20 μm. Phase diagram showing droplet formation by EGFP-nArgBP2 (**c**) and mEGFP-nArgBP2_959-1196_ **e** with PEG-8000 in vitro. Filled circles indicate that mEGFP-nArgBP2_959-1196_ formed liquid droplets via LLPS, while empty circles indicate that it did not. **f**–**j** COS7 cells were transfected with EGFP-nArgBP2_959-1196_ and imaged after 48 h. **f** Representative fluorescence images of droplets that underwent fusion over time (yellow arrowheads). Scale bars: 20 μm and 2 μm, respectively. **g** Time-dependent changes in the number and average size of droplets. *n* = 10 (0, 30, and 60 s) cells from three independent assays. Average droplet size, 0 s = 0.43 ± 0.09; 30 s = 0.50 ± 0.13; 60 s = 0.70 ± 0.17; values are means ± s.d. One-way ANOVA followed by Tukey’s HSD test. 0 s & 60 s ***p = 0.0003, 30 s & 60 s **p = 0.0070. **h** Representative time-lapse images showing fluorescence recovery after photobleaching EGFP-nArgBP2_959-1196_ droplets. Either round or cylindrical-shaped bleached areas were created. Scale bar: 2 μm. **i** Plots of normalized fluorescence intensity traces after photobleaching of multiple droplets. The average fluorescence intensity was traced as a thick line (*n* = 22). **j** Droplets are reversibly dispersed by 3% 1,6-hexanediol. Right: time-lapse images and kymographs of EGFP-nArgBP2_959-1196_ droplets are indicated by yellow rectangles in the low-magnification image. Scale bar: 20 μm. **k** Droplets of EGFP-nArgBP2_959-1196_ dissolved the addition of 10% 1,6-hexanediol (1,6-HD). (*Top*) The solution of purified EGFP-nArgBP2_959-1196_, which had been turbid due to phase separation, became clear after adding 1,6-HD. (*Bottom*) Fluorescence images of purified EGFP-nArgBP2_959-1196_ with or without 1,6-HD. Scale bar: 20 μm. **l** Fluorescence images showing the effect of salt concentration on nArgBP2_959-1196_ droplet formation. Five micromolar purified mEGFP- or mCherry-tagged nArgBP2_959-1196_ protein was dissolved in 5% PEG-8000 buffer (25 mM HEPES [pH 7.4] with various concentrations of KCl). Elevated KCl weakens droplet formation via LLPS. Scale bar: 20 μm.

Recent studies have implicated LLPS in diverse cellular processes as a dynamic and reversible regulatory mechanism. It can confine signaling effectors within complexes, thereby spatiotemporally fine-tuning their activity^26–28^. The critical driving forces for LLPS include multivalent, low-affinity interactions of biomolecules that present a high local concentration^29^. Since nArgBP2 contains three SH3 domains and proline-rich domains (PRDs) in the C-terminal region^7^, we tested whether nArgBP2 could form LLPS-mediated biomolecular condensates via its SH3-PRD interactions. We first transfected EGFP-tagged full-length nArgBP2, its three SH3 domains (nArgBP2_959-1196_), and nArgBP2 without SH3s (nArgBP2_1-958_) into COS7 cells that do not endogenously express nArgBP2 (Fig. 3a). We found that the heterologous expression of full-length and nArgBP2_959-1196_ constructs but not nArgBP2_1-958_ into COS7 cells formed large spherical condensates, much brighter than the surrounding cytoplasm, indicating that the SH3 domain-mediated interaction is required for the condensate formation of nArgBP2 (Fig. 3a). Next, we found that EGFP-nArgBP2_959-1196_-5P5A, a mutant in which the 5 proline residues in the PRDs changed to alanines, thus cannot mediate the intermolecular interaction with SH3 domains, failed to form dimers or coassemble into droplets in COS7 cells (Supplementary Fig. 7a–d). Together, these results indicate that biomolecular condensate formation of nArgBP2 is mediated by multivalent intermolecular interactions between SH3 domains and the PRDs.

We next sought to examine whether nArgBP2 possessed the potential for phase separation in vitro by performing cell-free assays. Both purified EGFP-full-length nArgBP2 and mEGFP-nArgBP2_959-1196_ alone formed large spherical condensates that had phase-separated from the bulk solution (Fig. 3b, d). We established phase diagrams by performing the condensate formation assay with various concentrations and a crowding agent (5% PEG-8000). The number and size of phase-separated condensates increased in an EGFP-nArgBP2 and mEGFP-nArgBP2_959-1196_ concentration-dependent manner (Fig. 3c, e). We further showed that mEGFP- and mCherry-tagged nArgBP2_959-1196_ also formed condensates in COS7 cells (Supplementary Fig. 7e), and both condensates completely overlapped each other in vitro (Supplementary Fig. 7f), thus indicating that condensates are formed regardless of fluorescent tags and ruling out the possibility of EGFP-tag-mediated artifacts.

nArgBP2_959-1196_ condensates often mobilized and spontaneously fused upon encountering one another, forming larger condensates over time (Fig. 3f, g). Fluorescence recovery after photobleaching (FRAP) experiments showed that upon photobleaching, the EGFP-nArgBP2_959-1196_ puncta recovered up to ~70% of the initial value within a few seconds (Fig. 3h, i), indicating the liquid nature within condensates and a dynamic exchange with the surrounding cytoplasm. Furthermore, nArgBP2_959-1196_ condensates dissolved within a few seconds when exposed to 3% 1,6-HD. The condensates then reformed rapidly upon 1,6-HD removal (Fig. 3j). Experiments with purified mEGFP-nArgBP2_959-1196_ in vitro also showed that mEGFP-nArgBP2_959-1196_ condensates were dispersed with 1,6-HD (Fig. 3k). In addition, the number and size of condensates were reduced as the salt concentration was increased, indicating the involvement of, at least in part, ionic interactions (Fig. 3l). The nArgBP2_959-1196_ condensates thus displayed all characteristic features of LLPS in living cells as well as in vitro.

### Dispersion of nArgBP2 condensates by Ca^2+^/CaMKIIα-mediated phosphorylation

**Fig. 4:**
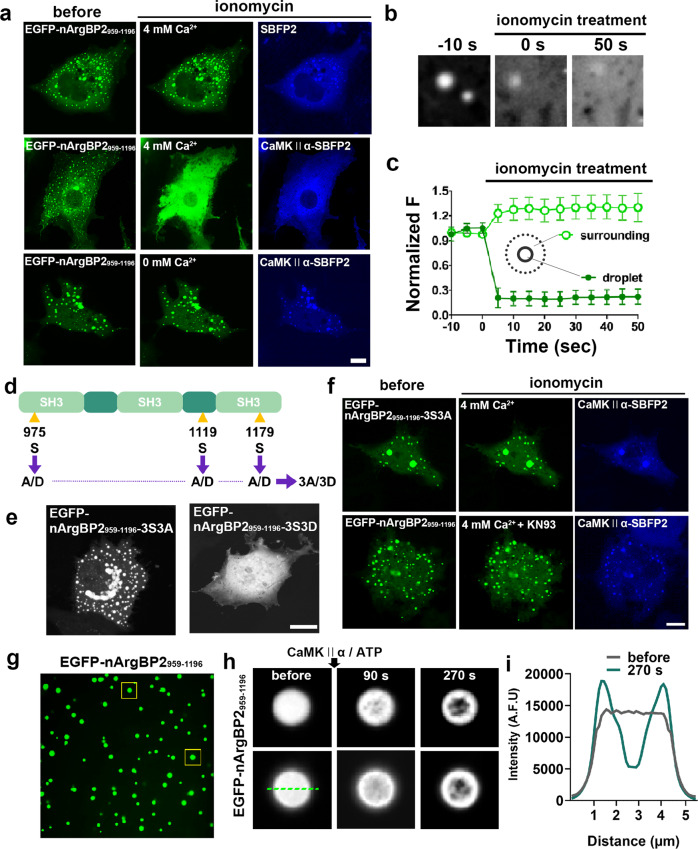
Biomolecular condensate coassembly of nArgBP2 with CaMKIIα and rapid dispersion by Ca^2+^/CaMKIIα-mediated phosphorylation. **a** Representative fluorescence images of COS7 cells transfected with EGFP-nArgBP2_959-1196_ and SBFP2 empty vector or CaMKIIα-SBFP2. EGFP-nArgBP2_959-1196_ droplets coexpressed with CaMKIIα-SBFP2 dispersed upon ionomycin treatment in 4 mM Ca^2+^ Tyrode (middle), while no effect on the droplets was observed without CaMKIIα coexpression (top) or in the absence of extracellular Ca^2+^ (bottom). Scale bar: 20 μm. **b** Enlarged time-lapse images of EGFP-nArgBP2_959-1196_ droplets coexpressed with CaMKIIα-SBFP2 in the middle panel of Fig. 4a before and after ionomycin treatment. Scale bar: 1 μm. **c** Plot of the normalized average fluorescence intensity profiles of EGFP-nArgBP2_959-1196_ droplets (filled circle) and the surrounding cytosol (open circle) over time. *n* = 54 for both droplets and surroundings. Values are means ± s.d. **d** Schematic diagram of nArgBP2_959-1196_-3S3D (phospho-mimetic) and nArgBP2_959-1196_-3S3A (phospho-deficient) mutants. **e** Representative fluorescence images of COS7 cells transfected with EGFP-nArgBP2_959-1196_-3S3D and EGFP-nArgBP2_959-1196_-3S3A. Scale bar: 20 μm. **f** Representative fluorescence images of COS7 cells cotransfected with EGFP-nArgBP2_959-1196_-3S3A and CaMKIIα-SBFP2 and treated with ionomycin or KN93 and ionomycin in the presence of 4 mM Ca^2+^. Scale bar: 20 μm. **g** Fluorescence images of purified EGFP-nArgBP2_959-1196_ before treatment with CaMKIIα and ATP. **h** Enlarged time-lapse images of purified EGFP-nArgBP2_959-1196_ (yellow squares in **g**) before and after treatment with CaMKIIα and ATP. Scale bar: 2 μm. **i** Line scan plots of EGFP-nArgBP2_959-1196_ (green dashed line in **h**) fluorescence intensity before and after CaMKIIα and ATP treatment.

We showed that CaMKIIα-mediated phosphorylation is required for the dispersion of nArgBP2 condensates in the spines of living neurons (Fig. 2). To further confirm this in the heterologous expression system, we coexpressed EGFP-nArgBP2_959-1196_ and SBFP2-tagged CaMKIIα in COS7 cells and found that they were coassembled into condensates. Upon activation of CaMKIIα by elevating cytosolic Ca^2+^ with ionomycin, however, the condensates underwent rapid and complete dispersion (Fig. 4a–c). No effect was observed in the absence of CaMKIIα or extracellular Ca^2+^ (Fig. 4a). The phospho-deficient nArgBP2_959-1196_-3S3A mutant also formed liquid droplets, but these droplets were not dispersed by ionomycin treatment, even when coexpressed with CaMKIIα (Fig. 4d–f), which is consistent with the results in living neurons (Fig. 2e, g). The phospho-mimetic nArgBP2_959-1196_-3S3D mutant in which three putative CaMKII phosphorylation sites were modified to aspartic acid (3D) failed to form condensates at all (Fig. 4d, e). The CaMKII inhibitor KN93 also abolished the dispersion of EGFP-nArgBP2_959-1196_ condensates by ionomycin (Fig. 4f). These results further support our findings that CaMKIIα-dependent phosphorylation is required for the dispersion of nArgBP2 condensates.

The purified nArgBP2_959-1196_ also formed liquid droplets, but upon treatment with CaMKIIα and ATP in the presence of calcium and calmodulin, these droplets were not dissolved, but the fluorescence translocated to the periphery, forming phase-in-phase assembly (Fig. 4g–i). This is reminiscent of the previous finding that CaMKII and GluN2Bc in the condensates move to the periphery, forming a distinct phase-in-phase assembly in a Ca^2+^/CaMKII-dependent manner in vitro^30^.

### WAVE1 binds to nArgBP2, and their interaction interferes with the phase-separating property of nArgBP2

**Fig. 5:**
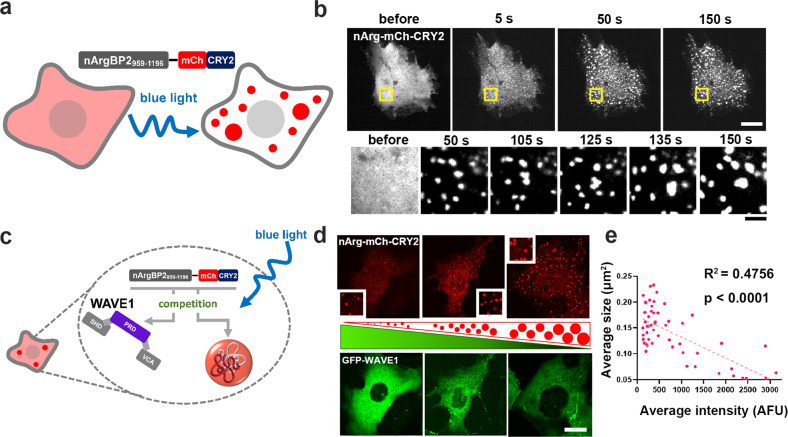
WAVE1 binds to nArgBP2, and their interaction interferes with the phase-separating property of nArgBP2. **a** Schematic figure of light-activated nArgBP2_959-1196_-mCh-CRY2. **b** Representative time-lapse fluorescence images of the light-activated assembly of nArgBP2_959-1196_-mCh-CRY2. COS7 cells were transfected with nArgBP2_959-1196_-mCh-CRY2 and stimulated with a 488 nm laser. Below: magnified images of the region enclosed by yellow rectangles in the low magnification images. Scale bars: 20 and 2 μm, respectively. **c** Schematic figure showing the interaction between WAVE1-PRDs and nArgBP2-SH3 domains competing with SH3-PRD-mediated self-oligomerization and phase separation of nArgBP2. **d** Representative fluorescence images of COS7 cells showing light-activated nArgBP2_959-1196_-mCh-CRY2 droplet formation at different levels of EGFP-WAVE1 expression. COS7 cells were cotransfected with nArgBP2_959-1196_-mCh-CRY2 and EGFP-WAVE1 and stimulated with blue light to induce nArgBP2_959-1196_-mCh-CRY2 droplets. Schematic triangular figures in the center illustrate the increase in the size of nArgBP2_959-1196_ droplets (red dots) and the decrease in WAVE1 expression levels (green). Scale bar: 20 μm. **e** Correlation between the relative fluorescence intensity (which reflects the expression level) of EGFP-WAVE1 and the average size of light-activated nArgBP2_959-1196_-mCh-CRY2 droplets. (*n* = 55, *R*^2^ = 0.4756, nonparametric Spearman correlation, *p* < 0.001).

We showed that nArgBP2 forms LLPS-mediated biomolecular condensates via its SH3-PRD interactions. Since the PRDs of WAVE1 also bind to the first and second SH3 domains of nArgBP2^7^, the interaction between WAVE1-PRDs and nArgBP2-SH3 domains might compete with SH3-PRD-mediated self-oligomerization and phase separation of nArgBP2 (Fig. 5c).

To test this hypothesis spatiotemporally, we took advantage of a photoactivated system to control phase transitions using the photolyase homology region (PHR) of *Arabidopsis thaliana* CRY2^31,32^. CRY2-PHR undergoes reversible homo-oligomerization and dissociation upon exposure to blue light and the absence of blue light, respectively (Fig. 5a). Therefore, we fused nArgBP2_959-1196_ to CRY2 and expressed nArgBP2_959-1196_-mCh-CRY2 in COS7 cells (Fig. 5a, b). We confined the analysis to early-transfected cells in which obvious droplets had not formed in advance. Upon exposure to blue light, nArgBP2_959-1196_-mCh-CRY2 formed distinct large fluorescent clusters (Fig. 5b). These clusters persisted after removal of the blue light, suggesting that nArgBP2_959-1196_-mCh-CRY2 formed condensed droplets (Fig. 5b). Then, nArgBP2_959-1196_-mCh-CRY2 and EGFP-WAVE1 were coexpressed in COS7 cells, and we analyzed the correlation between nArgBP2_959-1196_ droplet size and the level of WAVE1 expression (Fig. 5c–e). We found that the higher the levels of WAVE1 expression, the smaller the nArgBP2_959-1196_-mCh-CRY2 droplets formed upon blue light stimulation, suggesting that the interaction of nArgBP2_959-1196_ with WAVE1 interferes with the coacervation of cytosolic nArgBP2_959-1196_ into phase-separated droplets, resulting in smaller droplets (Fig. 5d, e). This is consistent with a previous study showing that WAVE1 is preferentially associated with monomers of ArgBP2^33^.

### Spine enlargement caused by nArgBP2 during cLTP is mediated by WAVE1 interaction

WAVE1 is known to direct signals through the Arp2/3 complex to regulate actin polymerization to manifest spine enlargement during cLTP^34,35^^,^ and we previously showed that nArgBP2 regulates spine morphology via WAVE-dependent pathways^7,12^. We thus hypothesized that the interaction between WAVE1 and nArgBP2 is required for spine enlargement during cLTP.

**Fig. 6:**
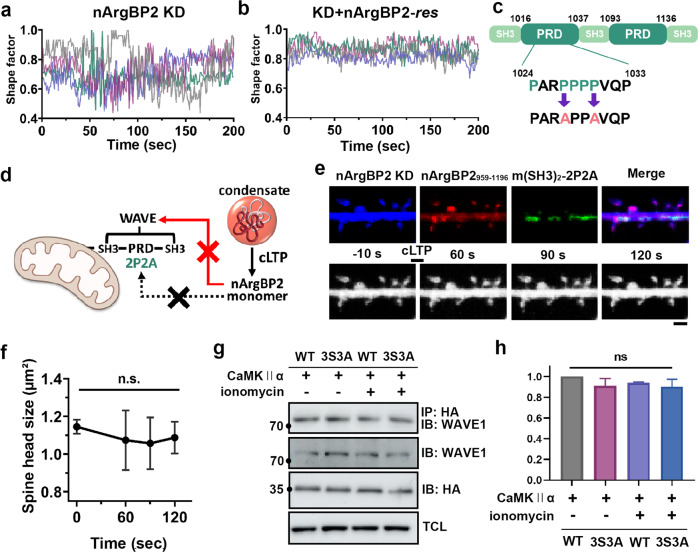
Spine enlargement caused by nArgBP2 during cLTP is mediated by WAVE1 interaction. **a**, **b** Hippocampal neurons were transfected with shRNA-nArgBP2 with or without nArgBP2-*res*. The shape factor of dendritic spines calculated from time-lapse recordings during cLTP. Changes in the shape factor, which is the index of spine motility, were analyzed with the form factor (sf = 4π*A*/*p*2) plug-in of Fiji. Representative shape factor profiles of nArgBP2 KD and KD + nArgBP2-*res* spines during cLTP. Each color indicates an individual spine. **c** Schematic diagram of mito-nArgBP2_959-1196_-2P2A, in which 2 proline residues (P1027 and P1030) were mutated to alanines (2P2A). **d**–**f** Hippocampal neurons were transfected with mTagBFP-tagged shRNA-nArgBP2, mCh-nArgBP2_959-1196_, and EGFP-mito-nArgBP2_959-1110_-2P2A to sequester WAVE from the cytosol to the mitochondria. cLTP was induced at DIV 21. **d** Schematic figure of the “knock-sideway” strategy using mitochondria-targeting SH3-1/2 (nArgBP2_959-1110_). **e** Representative fluorescence image of hippocampal neurons and time-lapse images upon cLTP. Scale bar: 2 μm. **f** The plot of time-dependent changes in single spine head size upon cLTP. One-way ANOVA followed by Tukey’s HSD test. *n* = 6, n.s., not significant. **g**, **h** COS7 cells were transfected with CaMKII and either wild-type or a phospho-deficient mutant (3S3A) of nArgBP2_959-1196_ and then treated with ionomycin. A coimmunoprecipitation experiment was performed to compare the interaction between nArgBP2 and WAVE1 before and after ionomycin treatment. **h** The plot of the relative intensity of the coimmunoprecipitation. One-way ANOVA followed by Tukey’s HSD test. *n* = 3, n.s., not significant.

To gain support for this hypothesis, we first measured the shape factor of spines, which indicates the stability of actin cytoskeletons^36^ (Fig. 6a, b). Previously, we demonstrated that nArgBP2 KD in developing neurons causes highly unstable actin dynamics^7,12^. We transfected shRNA-nArgBP2 with or without nArgBP2-*res* and then induced cLTP at DIV21. In KD spines, wide fluctuations in the shape factor were observed, suggesting the highly unstable nature of KD spines, while the shape factor value oscillates closer to 0.9 in KD + nArgBP2-*res*, indicating that spines constantly maintained their rounder shapes in these cells (Fig. 6a, b).

Next, we decided to block the interaction between WAVE1 and nArgBP2_959-1196_ during cLTP. We used the “knock-sideway” strategy using mitochondria-targeting SH3-1/2 of nArgBP2 that we have established in a previous study (Fig. 6d)^12^. Since the first and second SH3 domains of nArgBP2 bind to WAVE, we previously found that mitochondria-targeting SH3-1/2 successfully sequesters WAVE from the cytosol to the mitochondria, thus preventing WAVE from interacting with its binding partners^12^. In addition, to prevent oligomerization between mito-SH3-1/2 and nArgBP2_959-1196_, we used mito-nArgBP2_959-1110_-2P2A, a mutant in which two proline residues in the proline-rich sequences were changed to alanines (Fig. 6c). We expect that ArgBP2 is released from the condensates during cLTP, but the released ArgBP2 cannot interact with WAVE1 because it is sequestered into the mitochondria due to its interaction with mito-SH3-1/2 (Fig. 6d).

We transfected neurons with mTagBFP-tagged nArgBP2 shRNA, mCherry-nArgBP2_959-1196,_ and EGFP-mito-nArgBP2_959-1110_-2P2A and induced cLTP at DIV 21 (Fig. 6d–f). We found that blocking the interaction with WAVE1 completely abrogated the enlargement of the spine head upon cLTP (Fig. 6e, f). These results suggest that phase-separated nArgBP2 undergoes rapid CaMKIIα-dependent dispersion during cLTP, which in turn, together with WAVE1, manifests spine enlargement during synaptic plasticity (Supplementary Fig. 8).

Our results necessarily imply that phosphorylation of nArgBP2 inhibits its phase separation, but the interaction of phosphorylated nArgBP2 with WAVE1 should be maintained. We tested whether CaMKII-mediated phosphorylation of nArgBP2 affects binding to WAVE1 (Fig. 6g, h). We cotransfected COS7 cells with CaMKII and either wild-type or a phospho-deficient mutant (3S3A) of nArgBP2_959-1196_, treated the cells with ionomycin and performed a coimmunoprecipitation experiment to compare the interaction between nArgBP2 and WAVE1 before and after ionomycin treatment (Fig. 6g). We found no difference in the interaction of nArgBP2 with WAVE1 between wild-type and phospho-deficient mutant regardless of ionomycin treatment (Fig. 6h), thus confirming that the interaction between nArgBP2 and WAVE1 is not affected by CaMKII-mediated phosphorylation.

## Discussion

Recent studies have reported that the formation of postsynaptic densities, as well as presynaptic active zones, may involve phase separation-mediated biomolecular assemblies. SynGAP and Fragile X Mental Retardation Protein (FMRP) were found to undergo LLPS^37–40^. PSD-95, GKAP, Shank, and Homer in vitro can generate highly condensed assemblies via LLPS^41^. The clustering of transmembrane AMPA receptor regulatory proteins (TARPs) into the PSD is mediated via LLPS^23^. Wu et al*.* also demonstrated that purified RIM and RIM-binding protein (RIM-BP) undergo phase separation and form presynaptic active zone-like condensates with voltage-gated Ca^2+^ channels^23^. Synapsin 1 together with synaptophysin also forms phase-separated droplets^22,42^. Evidently, LLPS is an attractive mechanism for spatiotemporally modulating molecular processes in response to physiological stimuli, which is critical for orchestrating signal transduction within and between neuronal synapses.

The SH3 domains of nArgBP2/ArgBP2 bind signaling protein kinases, ubiquitin ligases, and proteins involved in the regulation of the actin cytoskeleton, such as dynamin, synaptojanin, WAVE isoforms, and WAVE regulatory proteins^7^, suggesting that the SH3 domains appear to confer most of the known functionality of nArgBP2. We also found that the SH3 domains of nArgBP2 sufficiently mediate the structural remodeling of dendritic spines^12^. In the phase-separation context, nArgBP2_959-1196_ behaves similarly to full-length nArgBP2 in the physiological context of living neurons and COS7 cells as well as in vitro. We, however, are aware that conclusions from such experiments should be taken with care, as the property of any single domain does not reflect a complete picture of a whole protein, and concrete conclusions will require novel approaches to more precisely estimate the in vivo features.

What we have observed in Fig. 4g is reminiscent of the phase-in-phase assembly previously observed with CaMKII and GluN2Bc^30^ (Fig. 4g, h). Unlike the in vitro results, however, in living fibroblasts, we did not find segregation into phase-in-phase assembly but rather rapid dispersion of droplets upon ionomycin treatment (Fig. 4a–c). One reason for this difference may be due to the availability of other interacting molecules in cells. Indeed, nArgBP2 interacts with various molecules to confer its functions to meet cellular demands. Therefore, the released nArgBP2 is taken up by interacting molecules in the cells, reducing the local concentration of free nArgBP2, thereby dissolving the droplets. In agreement with this context, we showed that the interaction between nArgBP2 and WAVE1 interferes with the phase-separating tendency of nArgBP2 (Fig. 5). We further showed that the interaction between nArgBP2 and WAVE1 is not affected by CaMKII-mediated phosphorylation. Since three putative CaMKII phosphorylation sites are in the first and third SH3 domains and second PRD domain (Supplementary Fig. 4) while nArgBP2 interacts with WAVE1 via the first and second SH3 domains^7^, the second SH3 domain remains intact during cLTP-mediated phosphorylation, so it can mediate the interaction of nArgBP2 with WAVE1 even in the phosphorylated state. WAVE1 is also known to be autoinhibited as a component of the WAVE regulatory complex (WRC) at rest, and upon activation of WRC by Rac1, WAVE1 can interact with actin to extend actin filaments^43^. These results suggest that nArgBP2 at rest remains dormant by being sequestered in liquid droplets and that WAVE1 also remains inactive by forming the WRC. The interaction between nArgBP2 and WAVE1 may not be realized until both are released by LTP. Upon activation, the two proteins now work together to manifest structural remodeling by coordinating actin polymerization in the spines (Supplementary Fig. 8). Thus, LLPS exhibits switch-like behavior for timely signal transduction during synaptic plasticity.

One of the remaining issues is whether nArgBP2 forms condensates in developing neurons as well and, if so, unlike in mature neurons, why ablation of nArgBP2 in the resting state causes such profound effects on dendritic morphology. We suggest two possibilities. First, although nArgBP2 forms condensates in developing neurons, the high spontaneous activity, and substantial structural remodeling during development skew its balance in the direction of either dissolution or solidification of condensates. Indeed, we found that nArgBP2_959-1196_ expressed in developing immature neurons does form condensates in spines, but they often appear to be solid aggregates rather than liquid droplets, showing only ~8% fluorescence reduction upon 1,6-HD treatment (Supplementary Fig. 9). Alternatively, nArgBP2 condensate formation can be regulated by different mechanisms in developing and mature neurons. In this respect, it is worth noting that two major neuronal CaMKII isoforms, α, and β, differ in cellular localization, spatiotemporal expression, and sensitivity to Ca^2+^ signals due to their different binding affinity for calmodulin^44–46^. Indeed, CaMKIIβ is expressed in the brain during early embryonic stages and development, whereas CaMKIIα predominates in juvenile stages up to maturity^47,48^. This leads to the speculation that CaMKIIα may act as a selective synaptic tag in mature neurons by phosphorylating nArgBP2 in response to Ca^2+^ influx during synaptic changes.

A previous study reported the results of electrophysiological analysis of LTP and LTD in a pan-*Sorbs2* KO mouse model^11^. The researchers performed whole-cell patch clamp recording in dentate gyrus (DG) granule cells in acute brain slices of 5-week-old KO mice. The hippocampal DG receives inputs from the entorhinal cortex via two major paths, the medial perforant path (MPP) and the lateral perforant path (LPP). Zhang et al. induced LTP/LTD at the outer one-third of the molecular layer in the DG, which is mainly innervated by LPP input from the lateral entorhinal cortex, and found that the properties of LTP and LTD were not altered in *Sorbs2* KO mice compared to WT mice^11^. Importantly, however, another study found that immature neurons (4- to 6-week-old mice) do not reliably potentiate LPP synapses but instead develop an increasingly greater capacity for LTP with age and neuronal maturity (over 3–4 months)^49^. These results suggest that LTP at LPP synapses is weak in immature neurons and progressively increases with cell age over the course of several months. In addition, LPP-DG synapses are known to usually express LTD or synaptic depression, and MPP-DG synapses mainly express LTP^50^. Since Zhang et al. used LPP synapses in young immature neurons (5 weeks old)^11^, this could be the reason why they failed to observe the effect on LTP in young *Sorbs2* KO LPP synapses. Notably, Zhang et al. ablated all *Sorbs2* isoforms, including nArgBP2; thus, a deficiency of all ArgBP2 isoforms in the nervous system, including glial cells, could modulate neuronal function and synaptic plasticity.

Finally, it will be of interest to determine whether nArgBP2 forms condensates with other PSD proteins and whether the formation of nArgBP2 condensates is affected by other PSD proteins. Phase separation-mediated regulation of the activity of these proteins may provide new opportunities for understanding the convergent mechanisms underlying multiple neuropsychiatric disorders with overlapping clinical manifestations.

## Supplementary information

Supplementary material

## Data Availability

The data used to support the findings of this study are included within the article and Supplementary material. Raw data that support the findings of this study are available from the corresponding author upon reasonable request.
