# Supplementary material for: Biomolecular condensate assembly of nArgBP2 tunes its functionality to manifest the structural plasticity of dendritic spines
Source: Exp Mol Med. 2023 Jan 4;55(1):108–19. doi: 10.1038/s12276-022-00918-6 (PMC9898538; doi:10.1038/s12276-022-00918-6)
Supplement: Supplementary file 1 — Supplementary material [file 12276_2022_918_MOESM1_ESM.pdf]

Supplementary Information

Supplementary Fig. 1 Sustained expression of nArgBP2 during neuronal development, and confirmation of knock-down efficiency by nArgBP2 shRNA.

Supplementary Fig. 2 Expression levels of NMDA receptors, GluN1 and GluN2 were not altered with nArgBP2 KD in hippocampal neurons.

Supplementary Fig. 3 Expression of nArgBP2-*res* in ArgBP2 KD neurons showed similar expression levels to endogenous nArgBP2 levels.

Supplementary Fig. 4 Conservation of putative CaMKII phosphorylation sites across species, and the schematic figure of CaMKII phosphorylation-deficient mutant (3S3A).

Supplementary Fig. 5 nArgBP2$_{959\text{-}1196}$ droplets in living hippocampal neurons exhibited liquid-like properties.

Supplementary Fig. 6 Endogenous nArgBP2 formed liquid-like condensates in spines that were dispersed by 1,6-hexanediol treatment.

Supplementary Fig. 7 Multivalent intermolecular interactions between SH3 domains and proline-rich domains are responsible for the phase-separating behavior of nArgBP2.

Supplementary Fig. 8 Model for the role of nArgBP2 in enlargement of dendritic spines during LTP.

Supplementary Fig. 9 nArgBP2$_{959-1196}$ also formed droplets in developing neurons but these droplets are reluctant to be dissolved by 1,6 hexanediol treatment.

Supplementary Table. 1 Antibodies used in this study.

Supplementary Figure 1

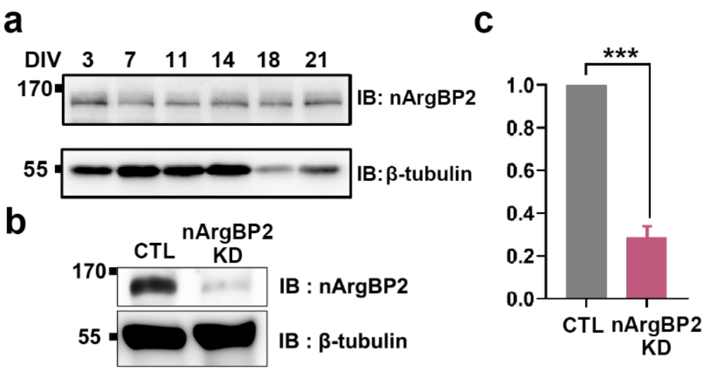

Supplementary Fig. 1 Sustained expression of nArgBP2 during neuronal development, and confirmation of knock-down efficiency by nArgBP2 shRNA. a Lysates from different developmental stages (DIV 3 – 21) were analyzed by Western blotting with anti-nArgBP2 antibody. β-tubulin served as a loading control. **b-c** Hippocampal neurons at DIV4 were infected with adeno-associated virus (AAV)-shRNA-nArgBP2 and the KD efficiency was confirmed by Western blotting with the anti-nArgBP2 antibody at DIV21. β-tubulin served as a loading control. Student's *t*-test, n=3.***p<0.001.

Supplementary Figure 2

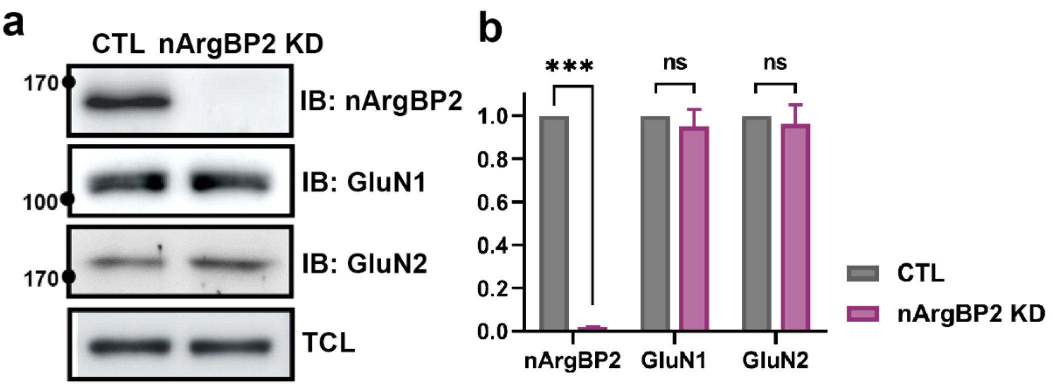

Supplementary Fig. 2 Expression levels of NMDA receptors, GluN1 and GluN2 were not altered with nArgBP2 KD in hippocampal neurons. a Hippocampal neurons at DIV14 were infected with adeno-associated virus (AAV)-shRNA-nArgBP2 and lysed at DIV21.Western-blot was performed with anti-nArgBP2, anti-GluN1, and anti-GluN2. IB, immunoblot; TCL, total cell lysates. **b** Relative intensity of western blot from (a). Student's *t*-test, n=3, n.s, not significant. ***p<0.001.

Supplementary Figure 3

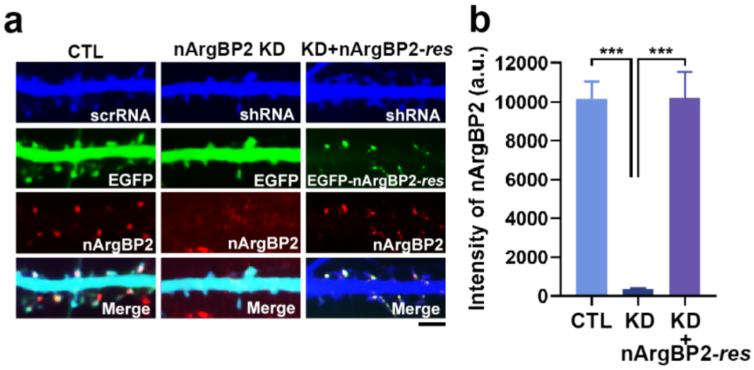

Supplementary Fig. 3 Expression of nArgBP2-*res* in ArgBP2 KD neurons showed similar expression levels to endogenous nArgBP2 levels. a Representative images of dendrites of neurons co-expressing the scrambled-shRNA (scrRNA) with EGFP (left), shRNA with EGFP (middle), and with EGFP-nArgBP2–*res* (right) respectively. res, a silent mutation that is resistant to shRNA. Scale bar: 10 μm. **b** The intensity of nArgBP2 from (a) showing with EGFP-nArgBP2–res co-transfected with shRNA express at a comparable level with endogenous level. One-way ANOVA followed by Tukey's HSD test. ***p<0.001.

Supplementary Figure 4

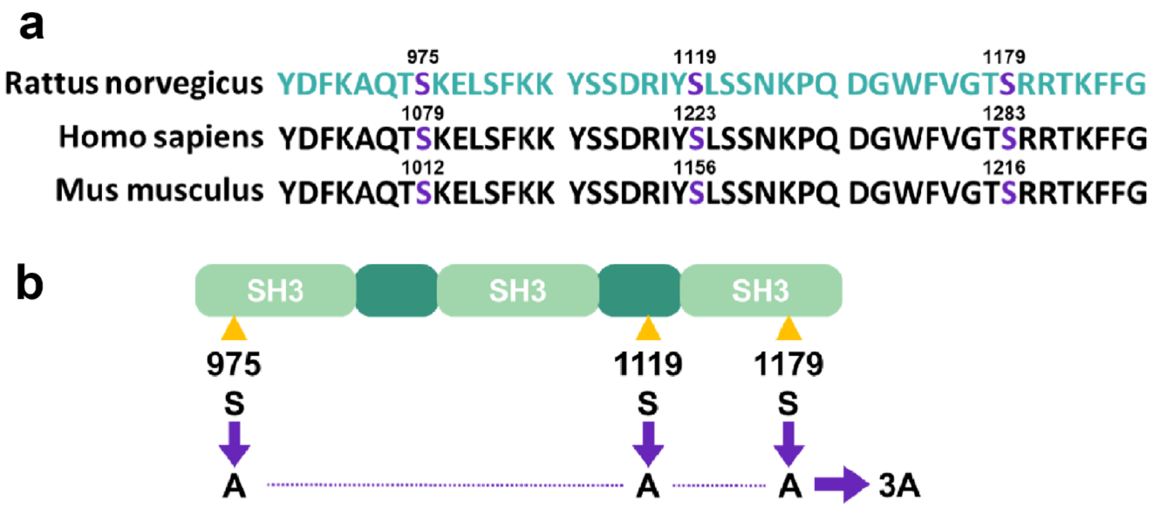

Supplementary Fig. 4 Conservation of putative CaMKII phosphorylation sites across species, and the schematic figure of CaMKII phosphorylation-deficient mutant (3S3A). a Multiple protein sequence alignment of nArgBP2 and orthologues from rat, human, and mouse that are conserved. **b** Schematic diagram of nArgBP2$_{959\text{-}1196}$-3S3A mutants.

Supplementary Figure 5

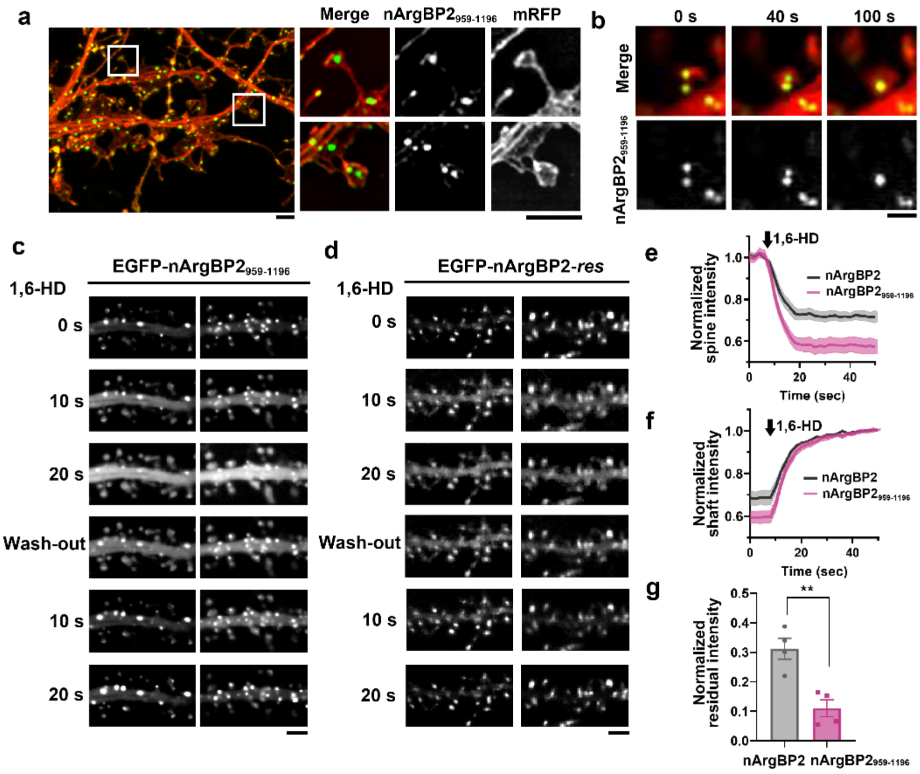

Supplementary Fig. 5 nArgBP2$_{959\text{-}1196}$ droplets in living hippocampal neurons exhibited liquid-like properties. a and b Hippocampal neurons were transfected with EGFP-tagged nArgBP2$_{959\text{-}1196}$ and mRFP-tagged shRNA-nArgBP2. **a** Representative images of nArgBP2$_{959\text{-}1196}$ condensates in dendritic spines. **b** Representative fluorescence images of droplets that underwent fusion over time. Scale bars: 2 µm and 1 µm, respectively. **c and d** Time-lapse images of hippocampal neurons transfected with EGFP-tagged nArgBP2$_{959\text{-}1196}$ **c**, or EGFP-nArgBP2-*res* **d** and mRFP-tagged shRNA-nArgBP2 upon treatment with, and removal of, 3% 1,6-HD. Hippocampal neurons were transfected at DIV 16 and imaged at DIV 21. Scale bar: 10 µm. **e-g** Plots of normalized fluorescence intensity traces of nArgBP2 and nArgBP2$_{959\text{-}1196}$ droplets in the spine **e** and shaft **f** after 1,6-HD treatment. **g** The normalized residual intensity of nArgBP2 and nArgBP2$_{959\text{-}1196}$ droplets after dispersion is complete (>30 sec). The fluorescence intensity of cytosol was subtracted from the intensity of the droplet. **p =0.0044, Student's *t*-test; Error bars indicate mean ± s.d.

Supplementary Figure 6

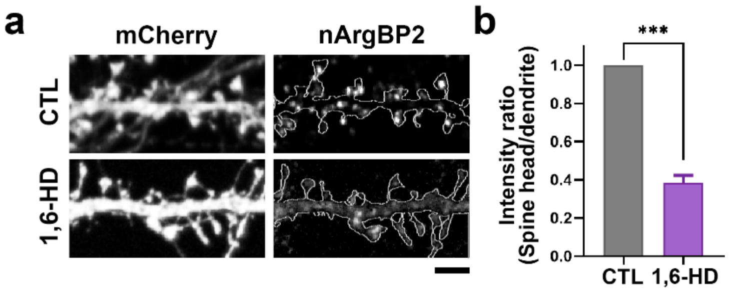

Supplementary Fig. 6 Endogenous nArgBP2 also formed liquid-like condensates in spines that were dispersed by 1,6-hexanediol treatment. a Hippocampal neurons were transfected with mCherry-C1 as a cytosolic marker and treated with 3% 1,6-hexanediol at DIV 21. Neurons were labeled with anti-nArgBP2. Scale bar: 2 μm **b** Plot of nArgBP2 intensity ratio (Spine head/ dendrite). ***p<0.00, Student's *t*-test; Error bars indicate mean ± s.d..

Supplementary Figure 7

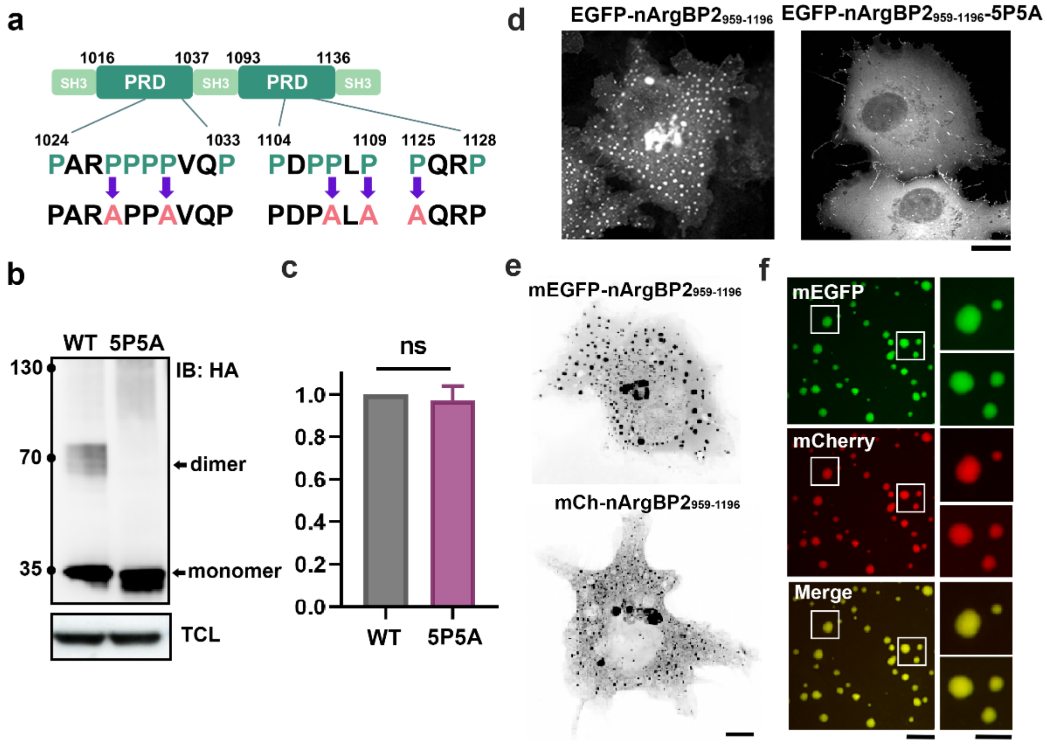

Supplementary Fig. 7 Multivalent intermolecular interactions between SH3 domains and proline-rich domains are responsible for the phase-separating behavior of nArgBP2. a Schematic diagram of nArgBP2$_{959\text{-}11963}$ construct showing the proline-rich sequences between SH3 domains. P1027, P1030, P1107, P1109, and P1125 were each mutated to alanines (5P5A). **b-c** Lysates from HEK293T cells transfected with HA-tagged nArgBP2$_{959\text{-}1196}$ (WT) or nArgBP2$_{959\text{-}1196}$-5P5A (5P5A) were treated with a cross-linking reagent (glutaraldehyde) and immunoblotted with anti-HA antibody. IB, immunoblot; TCL, total cell lysates. **c** Plot of the relative intensity of dimer and monomer of nArgBP2. Student's *t*-test; Error bars indicate mean ± s.d. n.s, not significant. **d** Representative fluorescence images of COS7 cells transfected with EGFP-nArgBP2$_{959\text{-}1196}$ or EGFP-nArgBP2$_{959\text{-}1196}$-5P5A. Scale bar: 20 µm. **e** Representative inverted images of mEGFP-tagged and mCherry-tagged nArgBP2$_{959\text{-}1196}$ expressed in COS7 cells. Scale bars: 10 µm. **f** Fluorescence images of purified mEGFP-nArgBP2$_{959\text{-}1196}$ and

mCherry-nArgBP2$_{959-1196}$ colocalized in droplets. 5 μM of each construct was mixed in a 1:1 molar ratio with 5 % PEG-8000. Scale bars: 20 μm and 10 μm, respectively.

Supplementary Figure 8

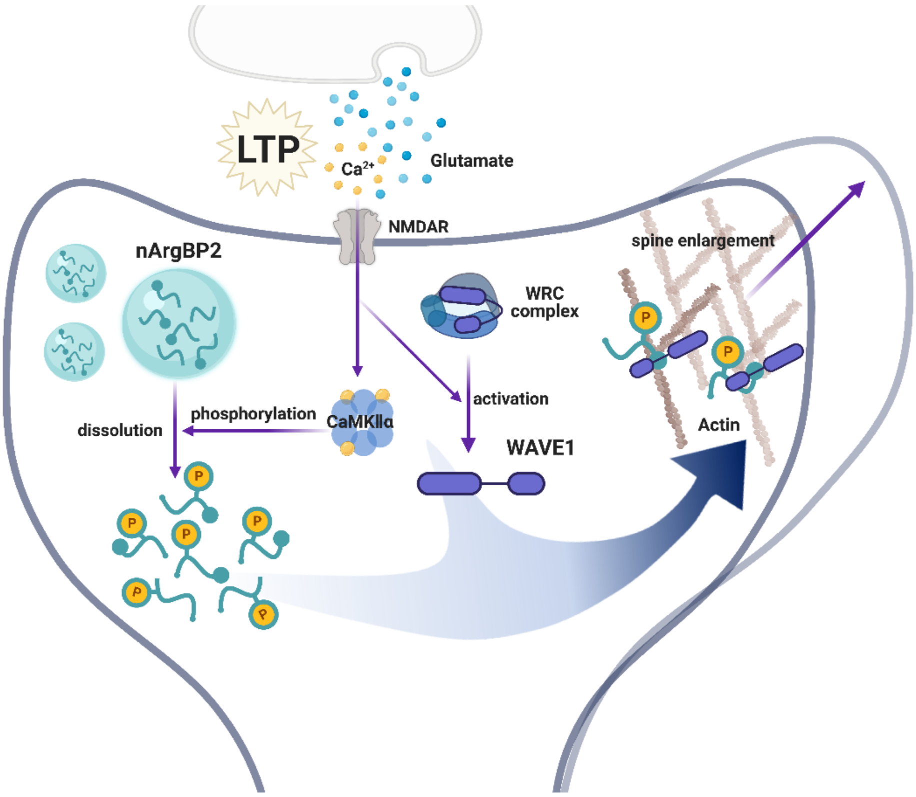

Supplementary Fig. 8 Model for the role of nArgBP2 in enlargement of dendritic spines during LTP. nArgBP2 at rest remains dormant by being sequestered in liquid droplets while WAVE1 also remains inactive by forming the WRC. Upon LTP induction, nArgBP2 is released from the droplets by calcium/calmodulin-dependent protein kinase II (CaMKII)-mediated phosphorylation, and WAVE1 is also released by the activated Rac1 and other kinases, thus two proteins now can interact with each other to regulate actin cytoskeletons and manifest the structural plasticity in dendritic spines. The schematic figure was created with Biorender.com.

Supplementary Figure 9

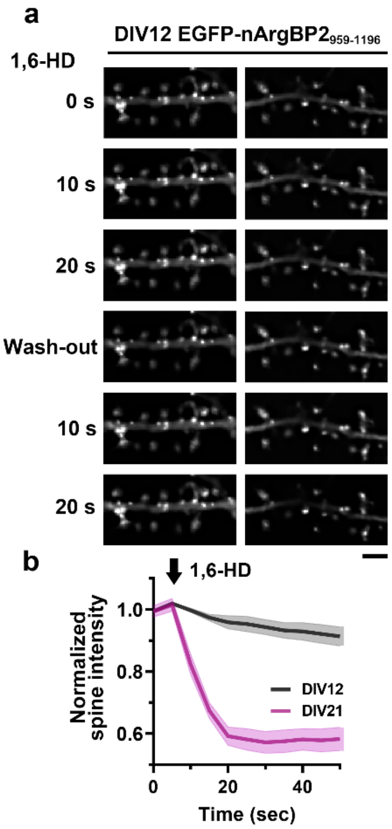

Supplementary Fig. 9 nArgBP2$_{959\text{-}1196}$ also formed droplets in developing neurons but these droplets are reluctant to be dissolved by 1,6 hexanediol treatment. a and b Hippocampal neurons were transfected with EGFP-tagged nArgBP2$_{959\text{-}1196}$ and mRFP-tagged shRNA-nArgBP2 at DIV7 and imaged at DIV12. **a** Representative time-lapse images of nArgBP2$_{959\text{-}1196}$ condensates upon treatment with, and removal of, 3% 1,6-HD. Scale bar: 10 μm. **b** Plots of normalized fluorescence intensity traces of nArgBP2$_{959\text{-}1196}$ droplets in DIV12 and DIV21 neurons after 1,6-HD treatment. The normalized residual intensity of DIV12 nArgBP2$_{959\text{-}1196}$ droplets after 40 s dispersion is 0.92±0.07. n=3

Supplementary Table. 1 Antibodies used in this study.

Antibody	Host	Company	Catalog number	Dilution
nArgBP2	Rabbit	Provided by Gianluca Cestra (Sapienza University of Rome, Rome)		1:500
SORBS2	Rabbit	Abcam	ab247000	1:500
GluN1	Mouse	Synaptic Systems	114 011	1:1000
GluN2 A/B	Rabbit	Synaptic Systems	244 003	1:1000
β-tubulin	Rabbit	Abcam	ab6046	1:1000
HA	Mouse	Convance	NMS-101R	1:1000
WAVE1	Rabbit	ECM Biosciences	WP1731	1:1000
Horseradish peroxidase-conjugated secondary antibodies	Rabbit	Jackson ImmunoResearch	111-035-003	1:5000
Horseradish peroxidase-conjugated secondary antibodies	Mouse	Jackson ImmunoResearch	115-035-003	1:5000
Alexa Fluor™ 568 secondary antibodies	Rabbit	Thermo Fisher Scientific	A11011	1:1000
Alexa Fluor™ 488 secondary antibodies	Rabbit	Thermo Fisher Scientific	A11070	1:1000
